# What Every Reader Should Know About Studies Using Electronic Health Record Data but May Be Afraid to Ask

**DOI:** 10.2196/22219

**Published:** 2021-03-02

**Authors:** Isaac S Kohane, Bruce J Aronow, Paul Avillach, Brett K Beaulieu-Jones, Riccardo Bellazzi, Robert L Bradford, Gabriel A Brat, Mario Cannataro, James J Cimino, Noelia García-Barrio, Nils Gehlenborg, Marzyeh Ghassemi, Alba Gutiérrez-Sacristán, David A Hanauer, John H Holmes, Chuan Hong, Jeffrey G Klann, Ne Hooi Will Loh, Yuan Luo, Kenneth D Mandl, Mohamad Daniar, Jason H Moore, Shawn N Murphy, Antoine Neuraz, Kee Yuan Ngiam, Gilbert S Omenn, Nathan Palmer, Lav P Patel, Miguel Pedrera-Jiménez, Piotr Sliz, Andrew M South, Amelia Li Min Tan, Deanne M Taylor, Bradley W Taylor, Carlo Torti, Andrew K Vallejos, Kavishwar B Wagholikar, The Consortium For Clinical Characterization Of COVID-19 By EHR (4CE), Griffin M Weber, Tianxi Cai

**Affiliations:** 1 Department of Biomedical Informatics Harvard Medical School Boston, MA United States; 2 Biomedical Informatics Cincinnati Children's Hospital Medical Center University of Cincinnati Cincinnati, OH United States; 3 Department of Electrical, Computer and Biomedical Engineering University of Pavia Pavia Italy; 4 ICS Maugeri Pavia Italy; 5 North Carolina Translational and Clinical Sciences Institute University of North Carolina at Chapel Hill Chapel Hill, NC United States; 6 Data Analytics Research Center University Magna Graecia of Catanzaro Catanzaro Italy; 7 Department of Medical and Surgical Sciences University Magna Graecia of Catanzaro Catanzaro Italy; 8 Informatics Institute University of Alabama at Birmingham Birmingham, AL United States; 9 Department of Informatics 12 de Octubre University Hospital Madrid Spain; 10 Department of Computer Science and Medicine University of Toronto Toronto, ON Canada; 11 Department of Learning Health Sciences University of Michigan Medical School Ann Arbor, MI United States; 12 Department of Biostatistics, Epidemiology, and Informatics Perelman School of Medicine University of Pennsylvania Philadelphia, PA United States; 13 Department of Medicine Harvard Medical School Boston, MA United States; 14 Laboratory of Computer Science Massachusetts General Hospital Boston, MA United States; 15 National University Health Systems Singapore Singapore; 16 Department of Preventive Medicine Northwestern University Chicago, IL United States; 17 Computational Health Informatics Program Boston Children's Hospital Boston, MA United States; 18 Clinical Research Informatics Boston Children's Hospital Boston, MA United States; 19 Institute for Biomedical Informatics University of Pennsylvania Philadelphia, PA United States; 20 Department of Neurology Massachusetts General Hospital Boston, MA United States; 21 Department of Biomedical Informatics Necker-Enfant Malades Hospital Assistance Publique - Hôpitaux de Paris Paris France; 22 Centre de Recherche des Cordeliers INSERM UMRS 1138 Team 22 Université de Paris Paris France; 23 Department of Computational Medicine & Bioinformatics University of Michigan Ann Arbor, MI United States; 24 Department of Internal Medicine Division of Medical Informatics University of Kansas Medical Center Kansas City, KS United States; 25 Section of Nephrology, Department of Pediatrics Brenner Children's Hospital Wake Forest School of Medicine Winston Salem, NC United States; 26 Department of Biomedical Informatics National University of Singapore Singapore Singapore; 27 Department of Biomedical and Health Informatics The Children's Hospital of Philadelphia Philadelphia, PA United States; 28 Department of Pediatrics Perelman School of Medicine The University of Pennsylvania Philadelphia, PA United States; 29 Clinical and Translational Science Institute Medical College of Wisconsin Milwaukee, WI United States; 30 See Acknowledgments

**Keywords:** COVID-19, electronic health records, real-world data, literature, publishing, quality, data quality, reporting standards, reporting checklist, review, statistics

## Abstract

Coincident with the tsunami of COVID-19–related publications, there has been a surge of studies using real-world data, including those obtained from the electronic health record (EHR). Unfortunately, several of these high-profile publications were retracted because of concerns regarding the soundness and quality of the studies and the EHR data they purported to analyze. These retractions highlight that although a small community of EHR informatics experts can readily identify strengths and flaws in EHR-derived studies, many medical editorial teams and otherwise sophisticated medical readers lack the framework to fully critically appraise these studies. In addition, conventional statistical analyses cannot overcome the need for an understanding of the opportunities and limitations of EHR-derived studies. We distill here from the broader informatics literature six key considerations that are crucial for appraising studies utilizing EHR data: data completeness, data collection and handling (eg, transformation), data type (ie, codified, textual), robustness of methods against EHR variability (within and across institutions, countries, and time), transparency of data and analytic code, and the multidisciplinary approach. These considerations will inform researchers, clinicians, and other stakeholders as to the recommended best practices in reviewing manuscripts, grants, and other outputs from EHR-data derived studies, and thereby promote and foster rigor, quality, and reliability of this rapidly growing field.

## Introduction

What should researchers and clinicians conclude about the recent high-profile retractions of COVID-19 studies based on electronic health record (EHR) data? It is impressive that two publications involving patients with COVID-19, one in The Lancet [1] and the other in the New England Journal of Medicine [2], were determined to be unsound and were retracted in less than 2 months from publication, as these journals’ review processes and quality checks are among the most rigorous in the world. Yet, upon closer inspection by those of us familiar with EHR-based research, there were many flaws to these studies involving data quality issues and a lack of transparency that should have been more readily identified during the peer and editorial review process. This is not to say that in-depth statistical analysis might not have eventually uncovered concerns but rather to point out incongruities and anomalies unique to EHR-based studies that should immediately raise concerns to experienced biomedical informaticians, much like an experienced contractor explaining to a homeowner why a competing bid is too good to be true.

In this viewpoint, we present six key questions that are necessary to consider when appraising EHR-based research, especially for research studies investigating the pandemic:

1. How complete are the data?
2. How were the data collected and handled?
3. What were the specific data types?
4. Did the analysis account for EHR variability?
5. Are the data and analytic code transparent?
6. Was the study appropriately multidisciplinary?

In particular, we focus on general aspects of these questions that are crucial to study and data quality and validity of and interpretability of the results and that are broadly applicable to many stakeholders, including researchers and clinicians, in order to optimize the review of submitted manuscripts, published studies, and grant applications containing preliminary data. These desiderata were compiled by the 96 members of the Consortium for Clinical Characterization of COVID-19 by EHR (4CE)—a self-assembled group of collaborating hospitals focused specifically on studying the clinical course of patients with COVID-19 using EHR-based data—most of whom are biomedical informaticians—across 7 countries. 4CE members were invited to contribute their specific key concerns to a shared checklist. This list was then pared down into a less technical list for a more general audience. We excluded those items that are generally considered to be good biostatistical practices (eg, manual review of sample data sets, detecting and understanding outliers [3,4]) to present EHR-specific concerns to a broad biomedical audience. We also excluded recommendations that are contained within the Reporting of Studies Conducted Using Observational Routinely Collected Health Data (RECORD) statement [5,6], which are not specific to EHR-derived data. Finally, we did not focus on the specific limitations of EHR-derived studies, which have been amply documented [7,8], or on the methods to minimize the impact of these limitations, as this viewpoint is not focused on reviewing specific methodological options for investigators using EHR-derived data, which has been reviewed in detail previously [9-11]. We acknowledge that there are many other criteria that can inform evaluations of EHR-based studies, but we have purposefully limited this discussion to those issues that are most relevant to a general audience, centered on studies investigating the pandemic.

## Data Completeness

There are several statistical tests to query data completeness and methods for incorporating missing data [12,13], but here we describe the reasonable expectations for such completeness with knowledge of current, state-of-the-art EHR usage. A publication that is specific about which data were obtained from the EHR (eg, specific laboratory tests or billing codes) is more credible than a study that simply claims it obtained 100% of the EHR data (as did the two recently retracted publications [1,2]). The range of data types from EHRs is extensive and highly varied; each data type requires its own specific quality control and transformations to standard terminologies. For example, laboratory measurements alone can have as many as hundreds of thousands of local codes at a large health care system such as the Veterans Health Administration. In many cases, these data require some level of manual record review to assure data quality and completeness.

Similarly, if a study reports a deidentification procedure, it must describe the details of said procedure. The goals of the deidentification process determine the nature of the deidentification process and the associated regulatory requirements. For example, US hospitals can meet HIPAA (Health Insurance Portability and Accountability Act) standards [14] if they require obfuscation of the counts of patients with rare clinical presentations below a specified prevalence threshold and if they employ date shifting. Knowledge of these methods is essential to analyzing and interpreting the derived data.

Some data types are represented theoretically in the EHR but in practice are only recorded occasionally. For example, standardized codes for smoking history or a family history of specific diseases exist but their underuse is well known. Thus, one cannot assume that the lack of smoking history codes equates to the patient being a nonsmoker. In such scenarios, one must provide an explicit description of the management of missing/null values. Many data elements, such as a complete pulmonary function test, exist in a fragmented form, scattered across different fields in the EHR, and are difficult to extract reliably. In addition, clinical notes allow clinicians greater qualitative expressivity on some of the above values, like smoking history, where they are documented more frequently but not consistently. The quality criteria for reporting narrative content from clinical notes are further addressed below.

Many clinical states are not represented explicitly in the EHR but can be inferred (often referred to as computational phenotypes). When a publication refers to hyperlipidemia, readers should ask themselves whether the hyperlipidemic phenotype is assessed from one or more lipid laboratory tests, billing diagnostic codes, prescription of lipid-lowering medication, or a combination of the above. It is important to document if only structured codes were used or if the phenotype was defined based on information extracted from clinical notes by using natural language processing (NLP) or manual chart review. Either a table describing these phenotypic methods or a reference to a public set of definitions (eg, Phenotype Knowledgebase, PheKB [15]) or a published algorithm with reported accuracy (as seen, for example, in Zhang et al [16] and Ananthakrishnan et al [17]) can provide transparency and precision to these EHR-driven computational phenotypes. The lack of this transparency should be a warning sign. If onset time or temporal trends of clinical events are used as outcomes, it is important to provide sufficient details on how the data were used to derive these outcomes, how granular time was incorporated (eg, by day, 24-hour period, or hour/minute), and to comment on their accuracy, since EHR data are particularly noisy with regards to capturing the timing of events [18,19].

If one uses EHR data to obtain population estimates (eg, prevalence of a complication per 100,000 patients), then additional information should be provided so that readers can determine which subset of patients from that population a given hospital’s EHR can capture. For example, if the EHR captures a patient’s hospitalization for heart failure, will the EHR also capture the preceding or subsequent outpatient clinic visits related to that hospitalization? With health maintenance organizations, such as Kaiser Permanente, that is much less of a concern, but many hospitals operate in a patchwork system where the patient’s data are spread across multiple heterogeneous EHRs that do not necessarily communicate. In our recent COVID-19 study [20], we found many instances in which patients with COVID-19 were transferred from another hospital; unless that other hospital was part of our consortium, it was impossible to have a complete record of their COVID-19 clinical course. It is also important to recognize that a given EHR may not fully capture the clinical course of certain patients, such as those infected with SAR-CoV-2 who have mild symptoms and are discharged home from the emergency room. In these instances, integration of EHR data with data from other sources (eg, primary care providers’ offices or nursing homes) may increase the reliability of analysis, although in practice this is rare and such integration methods have to be well documented. EHR systems may also fail to capture acute events that occur outside of the system, especially in the coded data. Leveraging NLP data from the clinical notes can potentially recover partial information if the patient has follow-up visits within that particular system.

## Data Collection and Handling

Often the units of measurement and the codes used for data elements like laboratory tests, medications, and diagnoses are not the same across hospitals and may even differ within the same health care system or change over time. Single analytic concepts (eg, the troponin T test) can balloon into dozens of local codes at each hospital, since these tests may be performed at different diagnostic laboratories, each with its own distinct codes or with different technologies over time. Therefore, they have to be “harmonized,” or mapped, to agreed-upon standard terminologies and scales [21]. Even when they are the same, their meaning can differ based on population or practice differences (eg, which sensitive troponin test is used or which reference range defines a test result being normal, or in children rather than in adults, whose normative values often change across the age range) [7]. In both instances, readers should expect that the specific procedures for harmonization or site-specific semantic alignment are described adequately in the Methods section (or via supplementary materials). A summary of this process can become increasingly complex within the usual confines of a Methods section for multisite and international studies where, by necessity, the site-by-site variability is high.

## Data Type

There are large methodological divides and divergent ethical challenges between codified data (eg, discrete laboratory values such as serum glucose) and narrative text (eg, discharge summary) from which characterizations are obtained using NLP. While both data types have their own limitations, methods that incorporate both can greatly improve the sensitivity and/or specificity of the clinical characterizations and phenotyping of a group of patients. For example, signs and symptoms are often not codified discreetly or consistently (eg, not entered into the EHR’s Problem List) but are written in the clinical notes. Similarly, outpatient medication documentation in clinical notes does not necessarily represent accurately the medications that the patient is actually taking, but prescriptions entered into the EHR may. Combining both codified and NLP data can substantially improve sensitivity and/or specificity and ideally one should always use this complementarity [22-24]. For example, only about 10% of pregnant women with suicide ideation have related codes and vast majority of the cases are only documented in the notes [25]. However, the ability to extract NLP data and the accuracy of those data may be limited by each institution’s informatics infrastructure and expertise as well as local institutional review board (IRB) constraints. Furthermore, NLP application to clinical narrative text is relatively new and more prone to large variability in the quality of the obtained characterizations. Particularly in countries with different languages, the NLP techniques and their performance may vary widely. For this reason, readers should expect a reference to the specific NLP methods used and their performance characteristics on data of the sort that the study collected and analyzed. For example, if someone describes the use of an NLP approach on discharge summaries in intensive care units in Italy, but the provided citation was validated only for use in outpatient notes written in English, readers can be legitimately concerned about the accuracy and validity of the patient characterizations in that study. Furthermore, if a study claims very high accuracy, readers should expect a report (or citation of a report) that shows an expert review of the NLP method validated against a representative sample confirming the claimed performance.

## Robustness Against EHR Variability

Beyond any variation in human biology across countries and continents, different styles of practice, and how different reimbursement schemes influence styles of practice and use of EHRs, have a very large impact on the nature of EHR data. Therefore, a multinational study should at least acknowledge these differences as a limitation or explicitly attempt to account for them in the analyses. For example, in COVID-19–related research, it has become increasingly apparent that there is an association between patient race/ethnicity and their risk for acquisition of and complications from COVID-19. However, this association is much less detectable in EHR data, as, for example, it is mostly invisible in data from Europe because several countries forbid collecting self-reported race in the EHR. Even in the United States, the coding of different ethnicities or multiracial identification is not standardized. In addition, some countries have far more comprehensive primary care EHR data sharing, whereas others (like the United States) cannot aggregate data systematically and consistently across major health care centers.

## Transparency

In order to ensure patients’ rights to privacy, patient-level data can rarely be shared outside an institution. In many EHR-driven studies, the code to extract data from a source EHR can be protected by confidentiality agreements with the EHR vendor and is thus difficult to share. Nonetheless, the code or algorithm for creating the variables used for analyses should be provided even if the detailed data extraction procedures are not shared because of commercial restrictions. Running the code on synthetic data sets that follow a standard data model can demonstrate code functionality and facilitate code reuse [26]. The code used to conduct statistical analyses and create visualizations—after data extraction—should also be shared in public repositories to enable other researchers to follow each step of the analysis and provide further transparency. While there are significant challenges to sharing patient-level data, one can share intermediate results and aggregate distributions to increase transparency and understand between-institution differences [27]. One should archive the data used for analyses, along with the associated data extraction codes, at the local institution to ensure reproducibility. Authors should also make the deidentified data available—either publicly in a repository or by request. While only a small fraction of readers typically look at the code, whether referenced on a file server or shared as supplementary methods, the availability of the code provides reassurance and validation that the study utilized proper methodologies.

## Multidisciplinary Approach

There may come a time when data can be aggregated automatically from multiple EHR environments to answer a particular question without relying on a human to understand the particular idiosyncrasies of each institution’s data and EHR system. Until that day, effective EHR data set analysis requires collaboration with clinicians and scientists who have knowledge of the diseases being studied and the practices of their particular health care systems; informaticians with experience in the underlying structures of biomedical record repositories at their own institutions and the characteristics of their data; data harmonization experts to help with data transformation, standardization, integration, and computability; statisticians and epidemiologists well versed in the limitations and opportunities of EHR data sets and related sources of potential bias; machine learning experts; and at least one expert in regulatory and ethical standards. Data provenance records should already exist to ensure compliance with privacy standards, so that authors can readily point to these processes and reference institutional officials who grant data access similarly to IRBs. In our experience, we often have an interdisciplinary team participate in the process of establishing the research question and study design, defining the data elements, and determining what analyses can be performed given the available data. It is also important that people with complementary skills work together to review and interpret the results [28]. Each of these steps is a major contribution deserving of authorship. Just as a population genetics study reporting across countries often has dozens of authors, so do we expect multihospital EHR-driven studies to acknowledge and name the individuals as authors and in doing so provide accountability for the dozens of procedures, checks, and balances necessary for the reliable extraction of EHR patient data. Consequently, contribution statements should list explicitly the responsibilities of each author with regard to study conceptualization and design, data extraction, data harmonization, data integration, data analysis, results interpretation, and regulatory and ethical oversight. Additionally, although reputation is sometimes overvalued, having *no* reputation or at least a track record of appropriate success should trigger greater attention to documenting the process to reach the same level of trust. Unlike a mathematical proof, simple inspection of the data may be insufficient and will become increasingly so in the era of data generated by machine learning algorithms purposefully built for the task of conditioning data to appear real. Trust and accountability become essential companions to transparency and clarity during the EHR analytic process.

## Conclusion

Similar to publications from the early days of the genomic revolution, which initially included extensive sections on DNA sequencing validation, methods, reagents, and conditions that became progressively briefer as trust was built and the methods commoditized, comprehensively and transparently reported methods of EHR data extraction and transformation are at least as important as subsequent statistical analysis and interpretation. We need to be open and transparent about the inherent limitations of the data and the analyses. We should also acknowledge alternative interpretations of the results (eg, outlier prescribing practices in one country that confound the apparent effects of that drug in that country). Extra caution is also needed in how we draw causal inferences from EHR data, especially given the noisiness and incompleteness of the data in addition to several sources of bias, though application of a causal model framework and specific causal inference methods may help mitigate some of these concerns. The recommendations we have outlined here (see Table 1 for our 12-item checklist) do not substitute for a durable research infrastructure that would enable tracking EHR data provenance along explicit source, ownership, and data protocols, which would allow for rigorous and routine quality assurance in the use of EHR data [29].

**Table 1:** 12-item checklist to assess electronic health record (EHR) data–driven studies.

Item	Reassuring	Concerning
Defining study cohort/data extraction	Reporting the precise definition of the domains and/or subsets of EHR data extracted for the study cohort and the information system sources	100% of the EHR said to be extracted or no specification of which subsets of the EHR data were obtained
Deidentification	Specific deidentification algorithm documented with acknowledgment of analytic consequences/limitations	Only a statement that deidentification was performed
Defining clinical variables/data type–specific omissions/limitations	For data types represented poorly in EHR codified data, either NLP^a^ is deployed on the EHR clinical notes or additional data sources (eg, self-reported questionnaires) are used. Procedures to deal with missing values should also be made explicit	Referencing data types like family/social history without explaining how they are obtained through NLP or exceptional codified data practice
Phenotypic transparency	Computational phenotypes that are more than just a specific native EHR variable (eg, hyperlipidemia vs a specific LDL^b^ measurement) are either defined in the study or a citation is given to algorithmic phenotype definitions	Clinical phenotypes are used in the study without specifying how they were derived from the EHR data
Generalizing EHR findings to the population/population denominator	Study heavily cautions on using prevalence/incidence estimates from the EHR data or refers to empirical estimates on how much of a patient’s entire health care is captured in that particular EHR	Direct estimates of prevalence or incidence from EHR frequencies without justifying that generalization
Data collection	Clinical forms or data models implemented in health care information systems are shared or clearly described. This includes the coding systems used	Mention structured data without specifying the clinical forms or data models. Mention coded data without mentioning coding systems
Data transformation/harmonization	Data transformation process shared or clear description of which methods were used to harmonize data to a standardized terminology, scale units, and account for different local usage	Mention of harmonization methods without specifying which ones and what problems were identified and addressed/overcome
Textual vs codified data	If textual data are used in the study, then specification of which clinical notes, in what language, with which NLP algorithm with either an explanation of or a citation to that algorithm’s validation, sensitivity, and specificity for comparable data	Harmonization efforts for codified and textual data treated as if they are the same process. Lack of specificity in describing the NLP algorithm and performance
Manual coding of data	Qualifications of coders described, formal coding criteria described or at least mentioned, intercoder reliability measured and reported	No description of process for turning text or nonstandard coded data into standard coded data; use of crowd-sourced coders (eg, graduate students or Mechanical Turk) without mention of quality assurance processes
Regional and global variation	A study describes how they adjust for (or exclude) differences that are due to variation in practice, regulation, and clinical documentation through the EHR from site to site	A study says they adjusted for regional or country differences in practice or EHR documentation but do not describe how they do it
Sharing analytic code	Analytic code is deposited in a public repository or study-specific public website	Code is not shared or only “shared on demand”
Acknowledge a multidisciplinary team	Authorships for all parts of the extraction-through-analysis pipeline with precision as to each contribution	Health care system sources not named or local health care system site collaborators not named

^a^NLP: natural language processing.

^b^LDL: low-density lipoprotein.

Finally, in crises such as the COVID-19 pandemic, we need to recognize that many studies can contribute to our understanding of what is happening to our patients and how our practices might affect patient outcomes. Overly generalized conclusions will likely strain the boundaries of what can be reasonably inferred from the kinds of data currently obtained through EHRs. Recommendations that flow from overly broad claims may irreversibly harm stakeholders, including patients and clinicians. Increased reader awareness of EHR-derived data quality indicators is crucial in critically appraising EHR-driven studies and to prevent harm from misleading studies, which will ensure sustainable quality in this rapidly growing field.

## Abbreviations

4CE: Consortium for Clinical Characterization of COVID-19 by EHR
EHR: electronic health record
HIPAA: Health Insurance Portability and Accountability Act
RECORD: Reporting of Studies Conducted Using Observational Routinely Collected Health Data
NLP: natural language processing
IRB: institutional review board
PheKB: Phenotype Knowledgebase
